# Supplementary material for: Pharmacokinomic Profiling Using Patient-Derived Cell Lines Predicts Sensitivity to Imatinib in Dermatofibrosarcoma Protuberans
Source: Cells. 2025 Jun 11;14(12):884. doi: 10.3390/cells14120884 (PMC12190883; doi:10.3390/cells14120884)
Supplement: Supplementary file 1 [file cells-14-00884-s001.zip › cells- 3666386 supplementary material Figures.pdf]

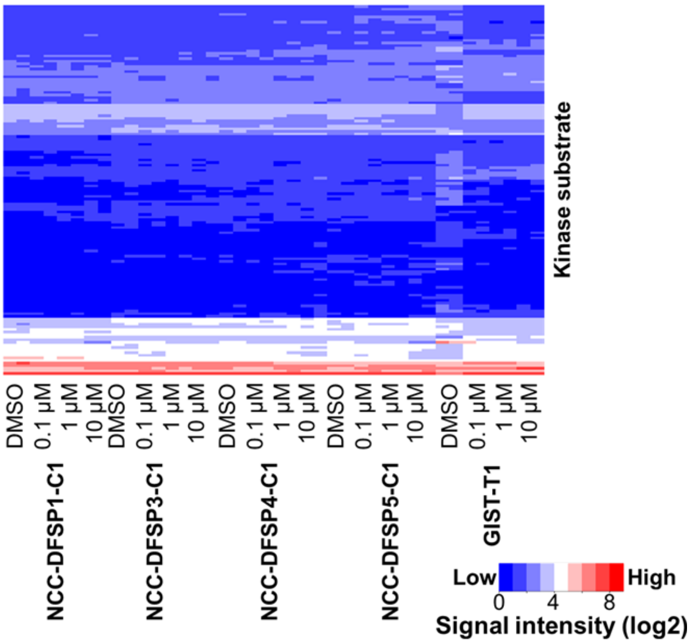

Figure S1: Kinase activity profiles of four DFSP PDCs and GIST-T1 cells

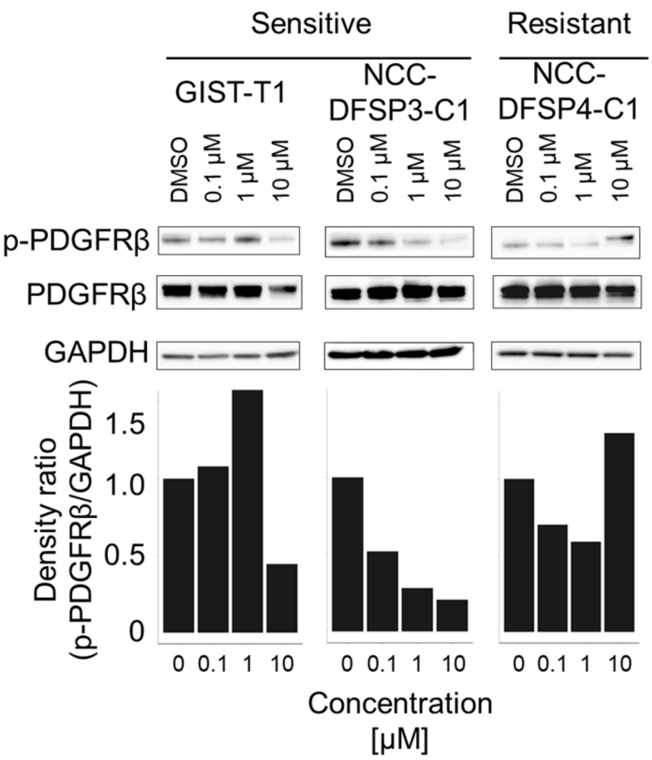

Figure S2: Comparative analysis of kinomic profiles and drug sensitivity data of imatinib between imatinib sensitive and resistant cell lines
